# Characterisation of the Cell Line HC-AFW1 Derived from a Pediatric Hepatocellular Carcinoma

**DOI:** 10.1371/journal.pone.0038223

**Published:** 2012-05-30

**Authors:** Sorin Armeanu-Ebinger, Julia Wenz, Guido Seitz, Ivo Leuschner, Rupert Handgretinger, Ulrike A. Mau-Holzmann, Michael Bonin, Bence Sipos, Jörg Fuchs, Steven W. Warmann

**Affiliations:** 1 Department of Pediatric Surgery and Pediatric Urology, University Children's Hospital Tübingen, Tübingen, Germany; 2 Department of Pediatric Pathology, University Hospital Schleswig-Holstein, Kiel, Germany; 3 Department of Pediatric Oncology/Hematology, University Children's Hospital, Tübingen, Germany; 4 Department of Medical Genetics, University of Tübingen, Tübingen, Germany; 5 Department of Pathology, University of Tübingen, Tübingen, Germany; University of Udine, Italy

## Abstract

Current treatment of paediatric hepatocellular carcinoma (HCC) is often inefficient due to advanced disease at diagnosis and resistance to common drugs. The aim of this study was to generate a cell line derived from a paediatric HCC in order to expand research in this field. We established the HC-AFW1 cell line from a liver neoplasm of a 4-year-old boy through culturing of primary tumor specimens. The cell line has been stable for over one year of culturing and has a doubling time of 40 h. The tumour cells have an epithelial histology and express HCC-associated proteins such as Alpha-fetoprotein (AFP), Glypican 3, E-cadherin, CD10, CD326, HepPar1 and Vimentin. Forty-nine amino acids in exon 3 of β-Catenin that involve the phosphorylation sites of GSK3 were absent and β-Catenin is detectable in the cell nuclei. Cytogenetic analysis revealed large anomalies in the chromosomal map. Several alterations of gene copy numbers were detected by genome-wide SNP array. Among the different drugs tested, cisplatin and irinotecan showed effective inhibition of tumour cell growth in a proliferation assay at concentrations below 5 µg/ml. Subcutaneous xenotransplantation of HC-AFW1 cells into NOD/SCID mice resulted in fast growing dedifferentiated tumours with high levels of serum AFP. Histological analyses of the primary tumour and xenografts included national and international expert pathological review. Consensus reading characterised the primary tumour and the HC-AFW1-derived tumours as HCC. HC-AFW1 is the first cell line derived from a paediatric HCC without a background of viral hepatitis or cirrhosis and represents a valuable tool for investigating the biology of and therapeutic strategies for childhood HCC.

## Introduction

Epithelial liver tumours, hepatoblastoma (HB) and hepatocellular carcinoma (HCC), are the most common primary hepatic malignancies in infants and children. HCC in children is less common than HB, accounting for approximately 1% of all paediatric cancers in the western hemisphere. In contrast to adults, most paediatric HCCs arise without liver abnormalities, although hepatitis, cholestasis, biliary athresia, glycogen storage disease, and low birth weight are risk factors for HCC development [1]. Several issues regarding paediatric HCC remain unresolved. Certain unique characteristics of paediatric HCC suggest a different biological origin and behaviour compared with adult HCC [2], [3]. Therapeutic results for children with HCC are generally poor despite a general increase in survival rates for most solid tumours among this age group. At present, the role of chemotherapy and the indication for liver transplantation in the treatment of paediatric HCC are critically debated [4]. In order to further address these issues preclinical models are essential. However, the establishment of cell lines and animal models for paediatric epithelial liver tumours is challenging and only a few HB cell lines have been successfully established during recent years [5]–[7]. There is currently no stable *in vitro* or *in vivo* model available for paediatric HCC. In this study, we describe the successful establishment of a continuous cell line derived from a child with HCC. The *in vitro* and *in vivo* model presented here might serve as tool for acquiring additional information and knowledge on this rare but important tumour entity.

## Methods

### Ethical statement

The study was done according to the ethical guidelines of the 1975 Declaration of Helsinki and written informed consent was obtained from the parents of the patient before operation. Ethics approval was obtained for this study from the ethic committee of the Medical Faculty of Tübingen. All animal studies were done according to the criteria outlined in the “Guide for the Care and Use of Laboratory Animals” (Animal Care and Use: Policy Issues in the 1990's, National Institutes of Health/Office for the Protection from Research Risks (NIH/OPRR). 1989. Proceedings of NIH/OPRR Conference, Bethesda, Md.), and were approved by the local Government's ethical authority for animal experiments (Regierungspräsidium Tübingen, Number CK1/09).

### Patient

The patient was a boy, 4 years and 6 months old, who presented with a large intra abdominal mass. No risk factors such as prematurity, viral infection, or developmental disorders were present at the time of diagnosis. Radiological assessment suggested a multifocal epithelial tumor in both sides of the liver. Multiple bilateral lung metastases were also identified, classifying the tumor as stage Pretext IV. Serum α-fetoprotein (AFP) at diagnosis was 400.000 µg/l. Treatment was initiated at this stage following the guidelines of the collaborative international center trial SIOPEL3. The high risk protocol consisted of 4× Cisplatin (CDDP, 80 mg/m^2^/24 h), 3× Carboplatin (CARBO, 500 mg/m^2/^/1 h), and 3× Doxorubicin (DOXO, 60 mg/m^2^/48 h). After these courses, the tumor showed stable disease with slightly decreased AFP levels and tumor volume. However, lung metastases had completely disappeared. After completing chemotherapy, a local progress (tumor volume) occurred. Consecutively, the patient received high dose chemotherapy (CARBO/VP16 according to the GPOH protocol HB99) together with autologous stem cell transplantation and a transarterial chemoembolisation. This led to a partial response of tumor volume and AFP (50.000 µg/l). With lung metastases still absent and the primary tumor being regarded as unresectable, the decision was taken to perform a living related liver transplantation (segments II and III) from the child's father. Immediately before operation, AFP rose again to a level of 153.000 µg/l, still there were no lung metastases detectable. Hepatectomy and liver transplantation were carried out 6 months after initial diagnosis.

### Tissue samples

Immediately after resection, primary tumor samples were shock frozen and stored in liquid nitrogen until use. Some tumor specimen were minced in PBS and cultured as described below.

### Cell lines and culture conditions

Primary tissue samples were minced into pieces of 3×3 mm and cultured on 6 well plates (Becton Dickenson, Frankfurt, Germany) in DMEM (GIBCO BRL, Carlsbad, CA) supplemented with 10% FCS (growth medium). Cell cultures were maintained in a humidified atmosphere containing 5% CO_2_ at 37°C. For sub-culturing cells were detached from the culture surface using accutase in Dulbecco's PBS containing 0.5 mM EDTA (PAA Laboratories GmbH, Cölbe, Germany) for 2–3 minutes at 37°C. A sub-cultivation ratio of 1∶4 and 1∶6 was performed twice per week. Cells were stored in liquid nitrogen as a suspension in complete growth medium with 10% DMSO.

### Viability assay

HC-AFW1 cells (10.000 cells/100 µl) were cultured in 96-well plates. At day two, the commercially available cytotoxic agents cisplatin (CDDP, Neocorp AG, Weilheim, Germany), doxorubicin (DOXO, cell pharm GmbH, Hannover, Germany), etoposide (Bristol-Myers Squibb GmbH & Co. KGaG, Munich, Germany), vincristin (Gry Pharma Kirchzarten, Germany), irinotecan (Fresenius Kabi AG, Bad Homburg, Germany), and carboplatin (Hexal, Holzkirchen, Germany) were added to the cells at different concentrations around IC_50_ (DOXO: 0.01, 0.03, 0.1, 0.3, 1.0, 3.0, 10.0 µg/ml; Carboplatin: 2, 4, 8, 16, 32, 64, 128 µg/ml; etoposide: 0.3, 1, 3, 10, 30, 100, 200 µg/ml; CDDP: 0.3, 0.63, 1.25, 2.5, 5.0, 7.5, 10 µg/ml; vincristin: 1, 10, 100, 1000, 10,000 100,000 ng/ml; irinotecan 0.78, 1.56, 3.12, 6.25, 12.5, 25, 50 µg/ml). Drugs were prepared immediately before administration, incubation lasted for 72 h. All assays were performed 3 times in quadruplicates. Cell viability was assessed using the MTT [3-(4,5-dimethylthiazol-2-yl)-2,5-diphenyltetrazoliumbromide]-assay. Percentages of viability were calculated through normalization between background of cultures without cells and untreated cultures as control experiments. Dose dependent viability curves were computed by sigmoidal curves with variable slope to determine IC_50_.

### Senescence

HC-AFW1 cells in the passage P5 and P20 were seeded at densities up to 5×10^4^ cells/cm^2^. The next day senescence was detected in cultures using the acid beta galactosidase staining (Cell signalling, Danvers, MA). Blue cells and unstained cells were counted in 6 different regions of triplicate cultures and percentages of senescent cells were calculated.

### Telomere length analysis

HC-AFW1 cells stored at passage P2 and P16 were processed for telomere length analysis using the flow FISH method [8]. As a reference, bovine leukocytes were used to calculate telomere length.

### Animal experiments

NOD.Cg-Prkdcscid IL2rgtmWjl/Sz (abbreviated NSG) mice were purchased from Charles River (Sulzfeld, Germany) and bred in our facility. Tumor cells were injected into the flank of 4- to 6-week-old mice (24–30 g), kept in filter-top cages at 22°C, 60% humidity. Sterilized food and water were accessible ad lib. HC-AFW1 cells (10^6^/200 µl/injection site) were injected subcutaneously. Tumor length (l) width (w) and height (h) were measured every 5 days. The tumour volumes (V = 4/3π×l/2×w/2×h/2) and mean diameter (V^1/3^) were calculated. Sigmoidal curves with variable slopes of the mean diameter were used to describe each tumor growth over 25 days. Blood samples were taken weekly from the retro-bulbar plexus of CO_2_/O_2_ – anaesthetized mice. Serum AFP levels were determined using a solid phase enzyme-linked immunosorbent assay (AFP ELISA assay; DRG Instruments GmbH, Marburg/Lahn, Germany), which was carried out according to manufacturer's protocol. Tumors were explanted on day 25 and prepared for further analyses.

### Histology and Immunostaining

Tumor specimen were fixed in formalin (3.7%) and processed for histological analysis. Tissue processing was continued in a vacuum tissue processor (Leica TP 1050, Leica Wiesloch, Germany). Sections of 5 µm were stained with hematoxylin and eosin. Immunhistochemistry in paraffin sections was performed using the ABC method as described previously [9]. For cryosections, tumor specimen were embedded in Tissue Tec O.C.T. ™ (Sakura Finetek, Alphen aan den Rijn, The Netherlands) and frozen in liquid nitrogen. Frozen specimens were sliced into 10 µm sections using a Leica Cryotom. Before staining, sections were fixed with methanol/acetone 1∶1 at −20°C for 10 minutes and then dried at room temperature. Slides were incubated in goat-serum (1%, DAKO, Hamburg, Germany) for 45 min to block unspecific binding areas. The used antibodies and the specific conditions are described in Table S1. Nuclei were counterstained with DAPI (4′.6-Diamidino-2-phenylindole dihydrochloride, 0.1 ng/ml, Sigma, Munich, Germany). Immunofluorescence microscopy was carried out on a Zeiss Axio Scope epifluorescence microscope (Carl Zeiss, Oberkochen, Germany) with a MRC5 camera. Images were processed using AxioVision 4.8.1 software. Staining of cultured cells was performed identical, except cells were grown overnight on cover gales coated with poly-D-lysine. Flow cytometry analysis was performed with trypsinized cells in FACS-buffer (PBS with 2% FBS, 2 mM EDTA, 0.005% NaN3; all Sigma-Aldrich, Munich, Germany) and the same antibodies as used in immunohistology. Data was acquired with FACSCalibur (Becton Dickinson, Heidelberg, Germany) and analyzed by FCS Express (De Novo Software, Los Angeles, CA, USA). Dead cells were excluded by 7-Aminoactinomycin D (7-AAD, BD Pharmingen™) staining.

### Detection of β-Catenin

Western blotting was carried out as recently described [10] using antibodies against β-Catenin (1∶1000; Zytomed (CAT-5H10), Berlin, Germany) and a stringent washing buffer (0.1% Tween20 in PBS). For mutation analysis genomic total RNA and DNA were prepared from cells, tumor tissue and EDTA-blood from the patient using RNease ans DNA extraction kit, respectively (Qiagen, Hilden, Germany). A 1688 bp fragment overlapping the exon 2 to 6 from CTNNB1 was amplified, sequenced and aligned to AY081165 [11]. Same primers were used in the RT-PCR to amplify a 831 bp gene fragment [12].

### CGH and cytogenetic analysis

Chromosome preparations from cultured cells and GTG-banding were performed using standard techniques. Fluorescence- in situ-hybridization was performed with subtelomeric probes (Vysis, Abbott GmbH, Wiesbaden Germany) for the chromosomes 1, 2, 3, 4, 5, 7, 9, 19, 20, 21, and 22 as well as a centromeric probe for chromosome 11 in order to verify some of the structural abnormalities. DNA from the patient's blood and tumor samples was isolated with the QiaAmp DNA mini Kit according to manufacturer's instructions (Qiagen, Hilden, Germany). Single nucleotide polymorphism (SNP) and copy number polymorphism (CNP) genotyping were performed at the Microarray facility of the University of Tübingen using the Genome-Wide Human SNP Array 6.0 and Genotyping Console™ (GTC) software (Affymetrix, Santa Clara, CA). Data were deposited on GEO (http://www.ncbi.nlm.nih.gov/gds?term=GSE29283, GSM723814).

### Statistical analyses

Data analysis was carried out using GraphPad Prism 4.00 (GraphPad Software, San Diego, Califonia, USA) and sigmoid dose response curves with variable slopes. All numeric data are expressed as means. Data plotted on graphs are means and SD. Significance was assumed for p<0.05.

## Results

### Primary tumour characteristics

**Figure 1:**
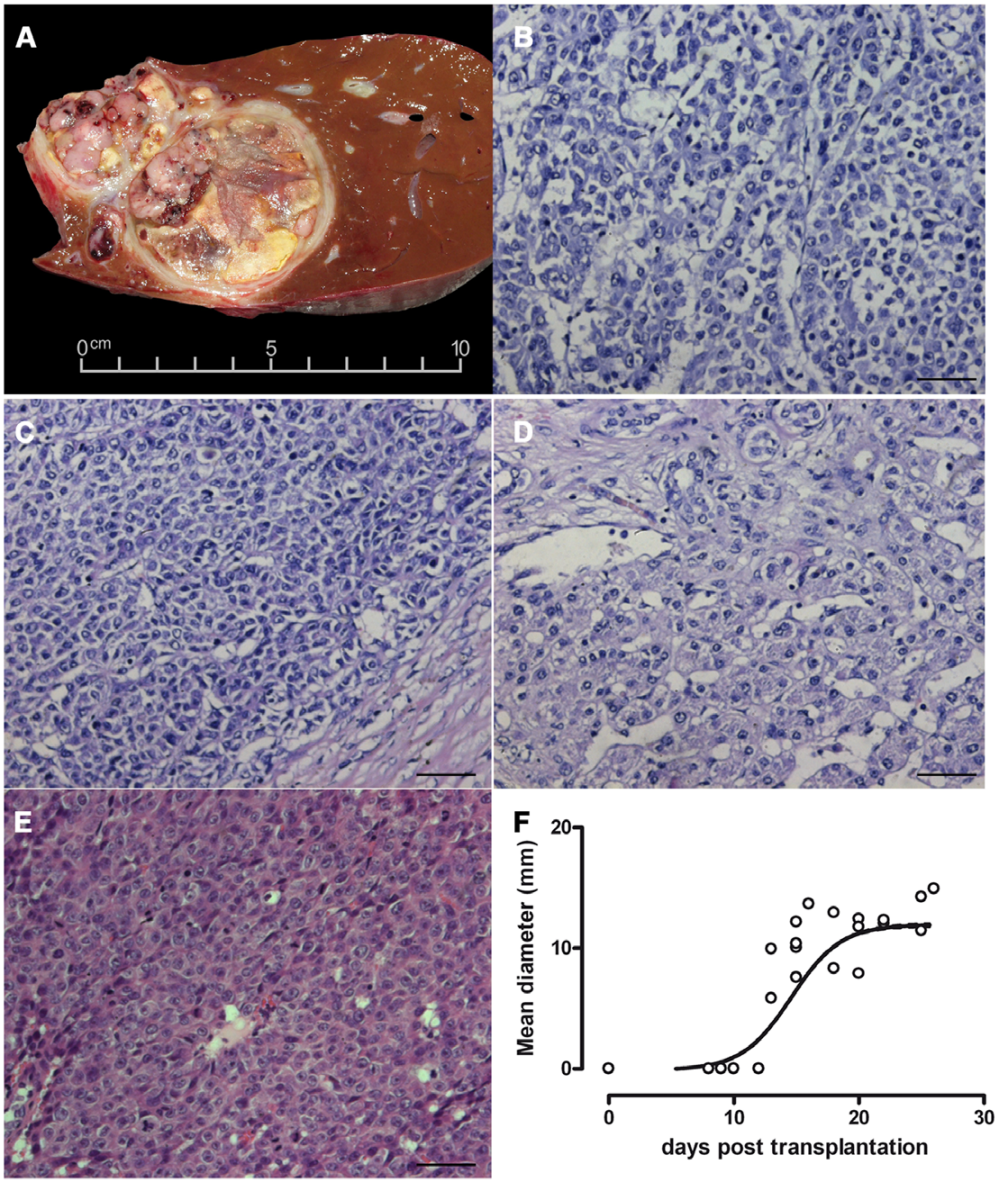
Histological appearance of the primary tumour and xenografts. Cross section of the explanted liver revealed multifocal lesions with a heterogeneous encapsulated tumour (A). Three areas of the primary liver tumour show epithelial and carcinoma-like cell morphology (B, C, D). Tumours generated in mice had a high cellular density (E). Tumour passages in mice led to tumour nodules growing exponentially in the second week after xenotransplantation, reaching a mean diameter of 12 mm within 10 days (F). Bars represent 50 µm.

Macroscopically, the tumour was characterized by multinodular, heterogenous areas with necroses. A cross section through the tumour and adjacent liver parenchyma revealed well circumscribed tumour nodules scattered throughout the non-cirrhotic liver with minimal macrovesicular steatosis (2%) and without fibrosis or cholestasis (Figure 1A). At the cut surface of the tumour was grey-yellow with large necrotic areas (40% of the tumour volume). Histological analysis revealed epithelial cells with carcinoma cell-type morphology (Figure 1B,C,D). Tumour nodules showed a solid macrotrabecular and focally pseudoglandular composition with polymorphic, polygonal, large eosinophilic tumour cells. The tumour cells had vacuolated polymorphic nuclei containing single large eosinophilic nucleoli. A large number of typical and atypical mitotic figures were seen. Typical features of fetal hepatoblastoma, heterologous elements, haematopoiesis, and mesenchymal components were not present. Routine histological staining revealed membrane-bound β-catenin in cells with nuclear localization in only a few distinct regions. P53 was not prominently expressed (data not shown). Glypican 3 and HepParI expression was strong and easily detected (Figure S1). The histological diagnosis at the time of surgery was HCC, which was confirmed by local and reference pathology (the latter performed by the GPOH study group) as well as by international expert review.

### Isolation of HC-AFW1 from native tissue

Two tumour specimens were used for tissue culturing and transplantation into NSG mice. Tumour cells were grown in culture from primary tumor samples and referred to as HC-AFW1. This cell line grows exponentially and has a doubling time of 40 h. Stable cell growth was observed for more than 19 passages over the last 12 months during which cytology, AFP secretion, and doubling time of the line were evaluated. Mice were injected with cultured cells after the 6^th^ population doubling. In mice the tumours grew within 4 weeks to a mean diameter of 15 mm. The tumours were transplanted continuously into new mice. Tumour xenografts displayed the same solid architecture as the primary tumour but contained slightly more pseudoglandular and fewer trabecular formations. The cells were polygonal with moderately large eosinophilic cytoplasm. The morphology of the nuclei was identical to that of the primary tumour cells, exhibiting vacuolization and prominent single eosinophilic nucleoli. The mitotic rate was high. No histological signs of further dedifferentiation (e.g. sarcomatoid pattern, giant cells) or features of HB were seen. Taken together, the histological analyses of the xenotransplants revealed the same characteristics as were observed in some regions of the primary tumour, which is consistent with a poorly differentiated solid HCC. Immunohistology revealed a predominantly nuclear distribution of β-catenin with membrane localization in only a few cells. The histological analysis of the xenotransplants revealed an appearance identical to that of the undifferentiated primary tumour (Figure 1E). HCC tumours grew exponentially to a mean diameter of 15 mm within the first 3 weeks after subcutaneous implantation and the tumors reached a plateau in the last observation week of monitoring (n = 6) (Figure 1F). Serum AFP could be detected before the subcutaneous tumour was apparent and the AFP level increased along with tumour development (Figure S2). Explanted tumour cells could be re-cultured on cell culture treated dishes.

### Genetic and phenotypic characterization of HC-AFW1 cells

**Figure 2:**
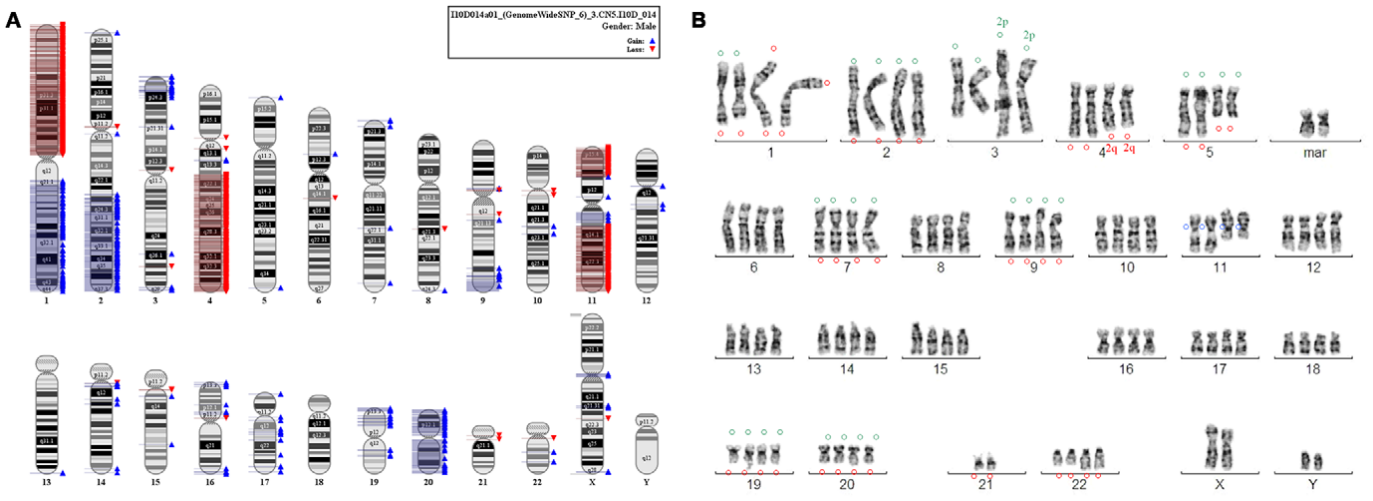
Cytogenetic of HC-AFW1 cell line. (A) Copy number analysis of the HC-AFW1 cell line is shown in the whole genome/chromosome view. Red flags denote loss and blue flags denote gain of copy number. (B) Karyotype of HC-AFW1. Green circles were used to mark the FISH signal of the short p-arm, red circles the long q-arm of the corresponding chromosome and blue circles the centromeric region of chromosome 11.

Chromosome analysis of HC-AFW1 cells revealed a mixture of cells with diploid and tetraploid karyotypes with several abnormalities (Figure 2). The detected structural and numerical aberrations seemed to be quite stable in different cells and there was no hint of mosaicism or clonal growth. In order to verify some of the structural abnormalities fluorescence-in situ-hybridization (FISH) with subtelomeric probes for chromosomes 1, 2, 3, 4, 5, 7, 9, 19, 20, 21 and 22 as well as a centromeric probe for chromosome 11 was performed. A tetraploid metaphase was selected because of good banding quality. Clearly visible were the interstitial deletion 1q, the isochromosome 1q, the derivative chromosome 3, the interstitial deletion 5q, a derivative chromosome 11, a marker chromosome (maybe a complex derivative chromosome 19), loss of 21, and duplication 22q. Additionally, a shorter derivative chromosome 4 was present. FISH analysis revealed der(4)t(2;4). A signal of 2p was present at the p-arm of the derivative chromosome 3, a 2q signal was detected at a C group-like chromosome—most probably at the shorter chromosome 4. There was also an additional signal of 5q at a D group chromosome that could not be further characterized. Table 1 summarizes the aberrations identified by cytogenetic analysis. These aberrations correlate with the results from the comparative genomic hybridization analysis. Comparison with published data on HB and HCC in the Atlas of Genetics and Cytogenetics revealed HC-AFW1 to be a unique entity (http://atlasgeneticsoncology.org/).

**Table 1:** Cytogenetic and array analysis of HC-AFW1 cells.

Hepatoblastoma*	Hepatocellular carcinoma*	Cytogenetic analysis and FISH of HC-AFW1	Array of HC-AFW1
Gain of 1	Loss of 1p (33%)Loss of 1q (22%)	Gain of complete 1q and loss of complete 1p by isochromosome 1q formationInterstitial deletion of 1q32–43	Gain of complete 1qLoss of complete 1p
Gain of 2qFrequent breaks in 2q35-q37		Gain of subtelomeric 2p: terminal 2p at 3pgain of subtelomeric 2q: der(4)t(2;4)(q36?;q31)	Gain of 2p25.3 (15 Mb)Gain of 2q24.1-qter
		Derivative chromosome 3 with terminal 2p at 3p and gain of unclear chromosomal material in 3q	Gain of 3pter-p24.3 (20 Mb) Gain of 3q29
Loss of 4q	Loss of 4q (38%)	deletion of 4q31-qterder(4)t(4;2)(q31;q36?)	Loss of 4q21.22-qter
		Interstitial deletion of 5q15–35.2Gain of terminal 5q (signal at a D-chromosome)	
	Loss of 6q (29%) [IGF2R]		No IGF2R deletion
Gain of 7			
Gain of 8	Loss of 8p (48%)		
	Loss of 9p (20%) [p16]		Gain of 9q33.1-qterNo p16 deletion
		Derivative chromosome 11 with unclear additional material in p and q	Loss of 11pter-p14.1Gain of 11p13.3-q12.2Loss of 11q13.4-qter
	Loss of 13q (31%) [RB1]		No RB1 deletion
	Loss of 16q (30%)Loss of 16p (24%) [Axin 1]		No Axin1 deletion
Gain of 17	Loss of 17p (45%) [p53]		No p53 deletion
			Gain of 19pter-p13.11
Gain of 20			Gain of complete 20
		Monosomy 21	
Gain of 22q		Duplication of 22q11.2–13	Gain of 22q12.1
t(1;4)(q12;q34) resulting in partial trisomy 1q and partial monosomy 4q		Additional marker chromosome, maybe a complex derivative of 19 with 20q at the short arm	

* According to the Atlas of Genetics and Cytogenetics in Oncology and Haematology.

**Figure 3:**
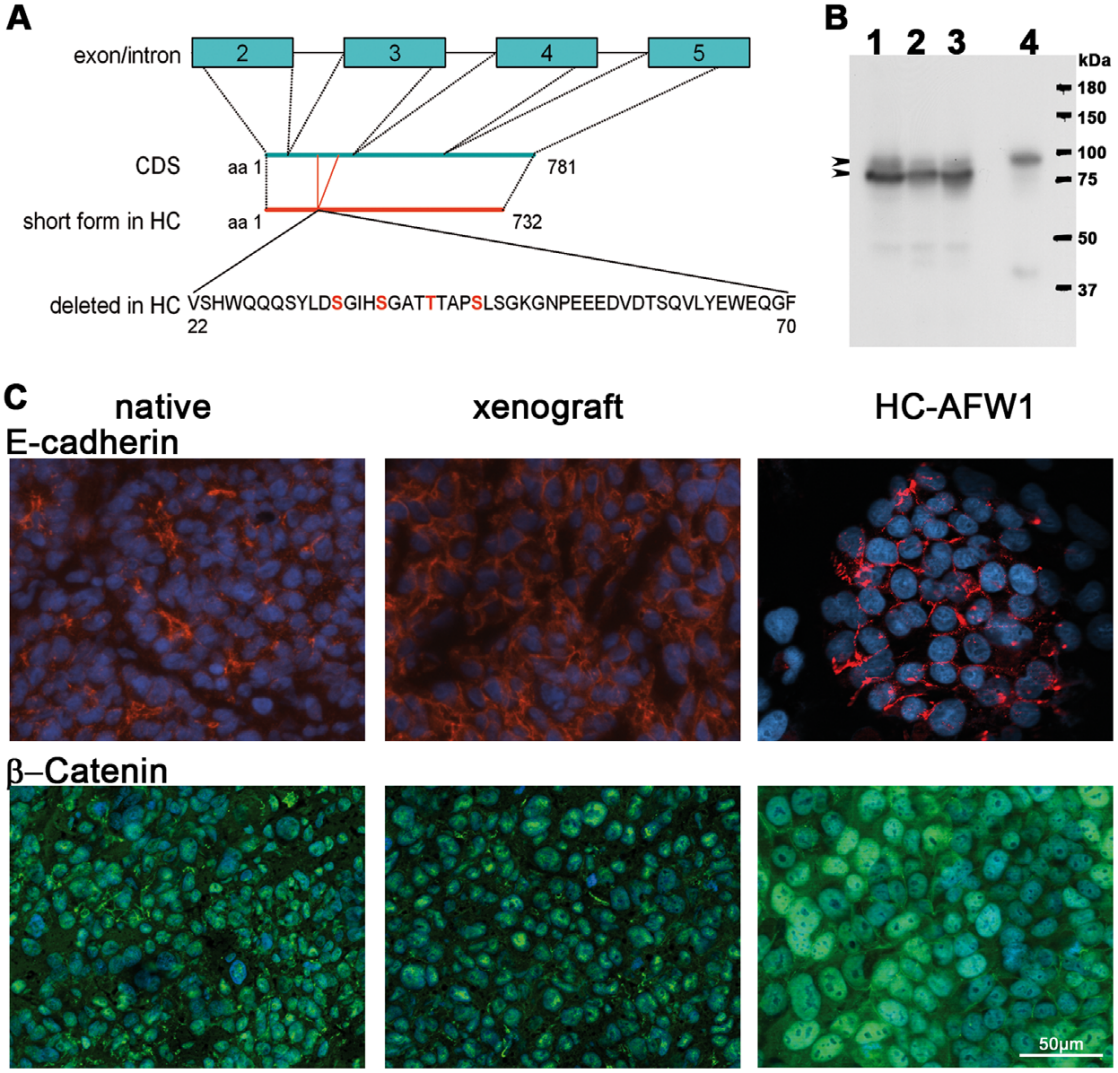
Expression of β-catenin and E-Cadherin in HC-AFW1 cells. (A) β-Catenin in HC-AFW1 cells revealed a deletion of a polypeptide from amino acids (aa) 22 to 70 coded on exon 3. In this region 3 serine residues and a threonine amino acid are depicted in red; as phosphorylation sites for GSK3. (B) Western blot analysis revealed a main short form of β-catenin in cultured HC-AFW1 cells (1), in xenografts (2) and in the primary tumour (3). A larger form was found in the native liver (4)(arrowheads). (C) E-cadherin in native and xenograft tissue and in cultured HC-AFW1 cells was located at cell-cell contact (red fluorescence). All specimen tumour cells showed green fluorescence staining for β-catenin in the cell nucleus, and in some cells a membrane localization. Cultured HC-AFW1 cells had notably stronger β-catenin staining in the nuclei. DAPI counterstaining indicates the cell nuclei (blue fluorescence).

The primary tumour and the established HC-AFW1 cell line were also screened for point mutations or deletions in exon 3 of the CTNNB1 gene encoding β-Catenin. PCR and RT-PCR analysis revealed 2 forms of β-catenin. Both PCR products were sequenced: The large form had no mutations (Figure S3). Sequencing data from the mutation analyses showed no mutations in CTNNB1; however, an extended deletion of 147 bp in exon 3 was detected in exon3, which led to the deletion of 49 amino acids (Figure 3). This deletion represents amino acids 22 to70 (AY081165.1 EMBL-Bank) and includes the phosphorylation sites Ser 33, Ser 37, Ser 45 and Thr41. In concordance, a shorter form of β-catenin was also detected in HC-AFW1 cells compared with liver cells by western blot. The deletion in β -catenin was present within the primary tumour and the derived cell line (after sequencing of exons 3–6). The western blot results confirmed the previously observed overexpression of the shorter form and the reduced expression of non-mutated β-catenin, as was expected from the RT-PCR and sequencing results. β-Catenin was detected in the cytoplasm but was predominant localized in the nuclei, as was revealed by the homogenous intense fluorescence detected during immunostaining of cultured cells and xenotransplants. In the primary tissue, heterogenous intense staining for β-catenin was also observed with areas of membrane and nuclear distribution. The cell adhesion molecule E-cadherin, which interacts with β-catenin to form cell adhesion sites, was detected on the cell membranes.

**Figure 4:**
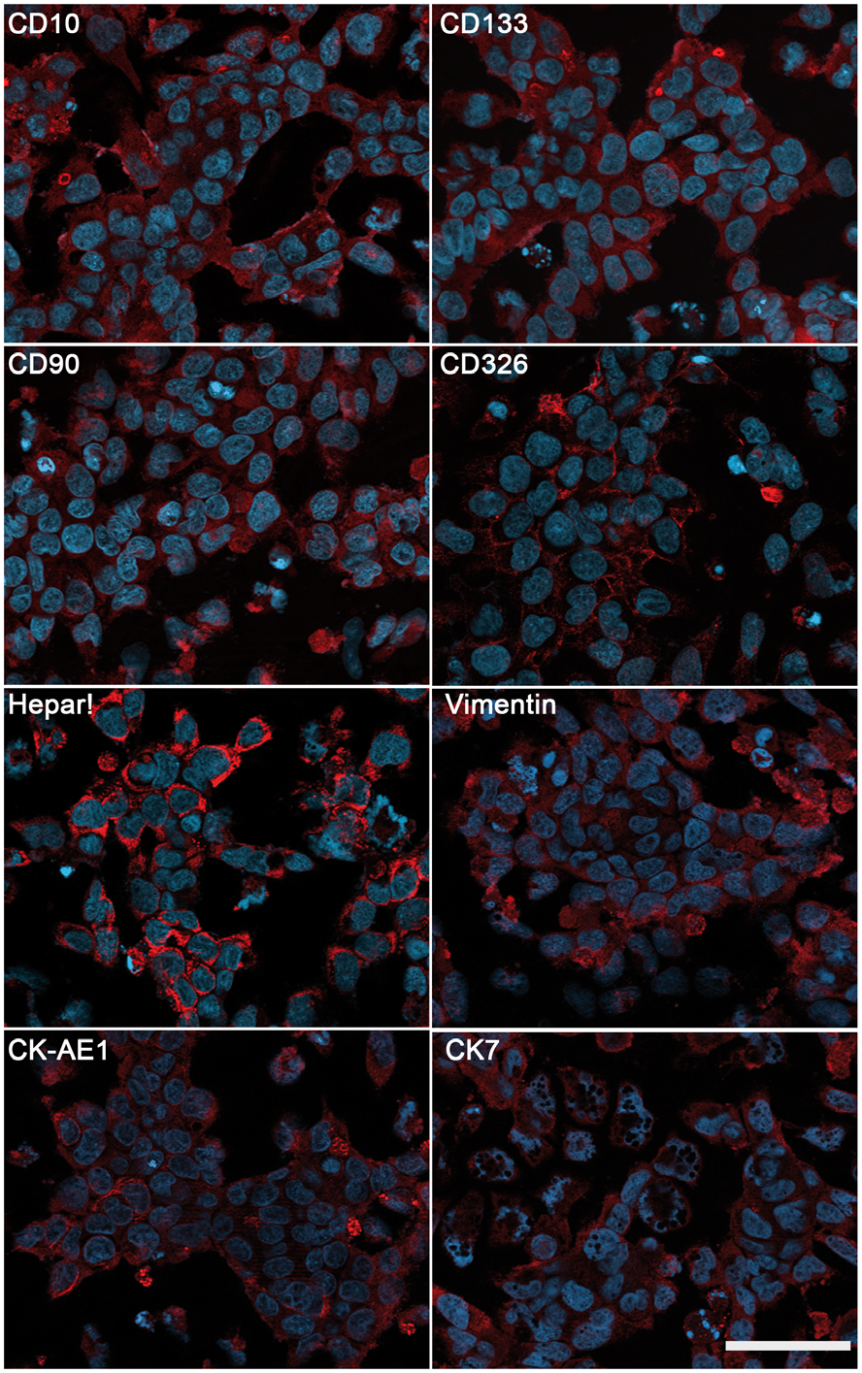
Expression of tumour-related proteins in HC-AFW1. Images show immunofluorescence staining of cultured cells. Red fluorescence denotes homogenous expression of CD90, CD326, cytokeratin type 1 (CK AE1) and HepPar1 as well as heterogeneous distribution of CD10, CD133, Vimentin and Cytokeratin 7 (CK7) within the cells. Blue fluorescence indicates nuclei (DAPI staining). Bars represent 50 µm.

**Figure 5:**
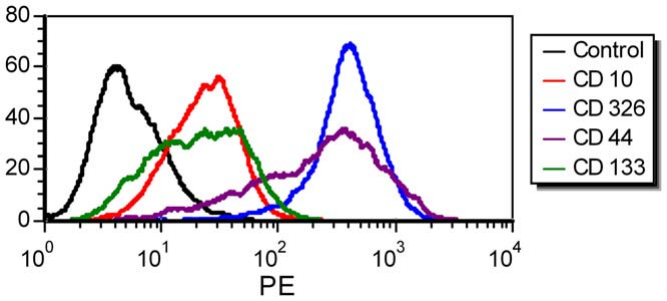
Expression of tumour markers on HC-AFW1 cells. Histograms from the flow cytometric analyses results revealed strong expression of CD326 and reduced expression of CD10 on HC-AFW1 cells. Staining for CD44 and CD133 showed a broad range of expression in the cell cultures. The isotype control fluorescence is plotted as a black line. Dead cells were excluded from the analysis by 7AAD staining.

AFP and Glypican 3 were detected in the original tissue, the xenotransplants, and in the cell line by routine histological staining. HC-AFW1 cells expressed AFP at a level of 34 IU/10^5^ cells at 24 h. Cultured cells showed membrane distribution of CD10, CD90, CD133 and CD326, as revealed by immunofluorescence (Figure 4). The antigen recognized by HepPar1 was present in the cytoplasm of all tumour cells. Vimentin was expressed in distinct areas where cells grew as 3D clusters. Cytokeratin 7 and cytokeratin type 1 (AE-1 clone) were expressed homogenously in the cell cultures and in the tumour tissue (Figure 4 and S1). Flow cytometry analysis of the HC-AFW1 cells revealed strong expression of CD326 on all of these cells. The cell culture was characterized by reduced expression of CD10 and by heterogeneous distribution of CD44, CD90 and CD 133 (Figure 5). Histograms of CD133 and CD44 staining revealed a broad peak, probably due to the presence of two distinct populations, as has been observed in most established cell lines [13].

To address the stability of the cultured cells the telomere length was estimated using the flow FISH technique. At passage 2, HC-AFW1 cells had a mean telomere length of 5.9 kb. At passage 16, the mean telomere length was 8.7 kb, which was also the length identified at passage 24 (Figure S4). Cell culture aging was assessed using acid beta galactosidase staining of senescent cells in cultures at lower (P4) and higher passages (P20 and P24). When the cells were plated at a high cell density of 5×10^4^ cells/cm^2^, less than 0.5% of the cells were senescent. At a lower plating density of 10^4^ cells/cm^2^, 25% of the cells at P4 were senescent. Only 11% of the cultured cells were senescent at the higher passages (Figure S4).

### Effect of cytostatic drugs on HC-AFW1 cells

**Figure 6:**
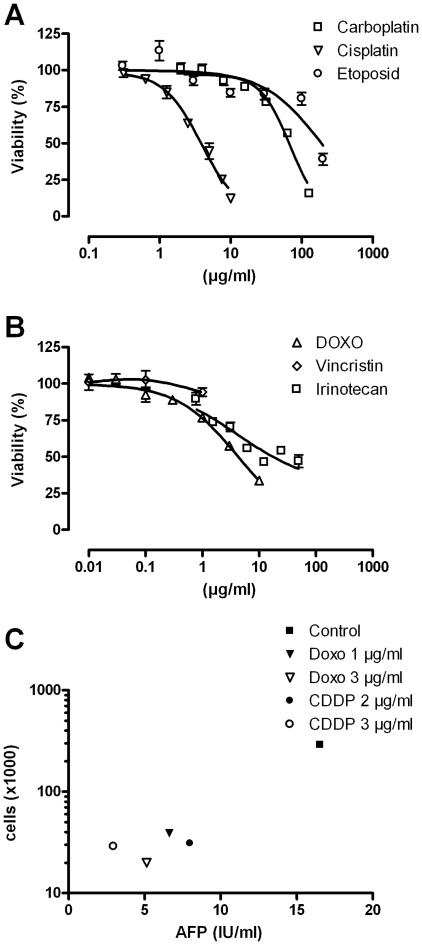
Sensitivity of HC-AFW1 cells to cytostatic drugs. Viability of HC-AFW1 cells treated with cytotoxic drugs. HC-AFW1 cells were incubated with cisplatin, carboplatin or etoposide (A) as well as vincristine, doxorubicin or irinotecan (B) at different concentrations. Relative cell viability was determined after 72 h by a cell viability assay. Means and SD from triplicate experiments are shown. Viable cells and AFP levels were determined in cultures treated with CDDP and doxorubicin for 48 h (C).

The HC-AFW1 cells were incubated with cytotoxic drugs at seven different concentrations in a viability assay. All drugs led to a marked decrease in the viability of the HC-AFW1 cells except for vincristine (Figure 6). The IC_50_ was 3.9 µg/ml for cisplatin, 68.3 µg/ml for carboplatin, 4.0 µg/ml for doxorubicin, 4.3 µg/ml for irinotecan and 190 µg/ml for etoposide. The response to cisplatin and doxorubicin was not significantly different among HC-AFW1 cells from different passages (P5 vs. P20). The AFP level in the culture dropped when HC-AFW1 cells were incubated with cisplatin and doxorubicin (Figure 6C). However, the AFP level was proportional to the rate of viable tumour cells, which was only 20% in treated compared to control cultures (determined by counting the live cells with trypan blue staining).

## Discussion

In this study, we describe the cell line HC-AFW1, as the first paediatric HCC cell line, which was not generated on the background of viral hepatitis or liver cirrhosis. This novel cell line presents histological and biological characteristics of an epithelial liver tumour. To date, continuous HCC cell lines have been generated exclusively from donors with alcoholic liver cirrhosis or hepatitis virus infection [14]–[16]. Based on the pathophysiology of the disease, these cell lines cannot serve as tools for investigating the biology of and therapeutic strategies for childhood HCC. Most paediatric HCCs in Europe are “de novo” cases, and are usually not related to hepatic cirrhosis [2]. The cell line HepG2 has been the focus of most attention. Initially, it was reported as a HCC. However, the authors later corrected their report and claimed that HepG2 was derived from a HB. HepG2 has been used in a variety of research studies focusing on metabolism, development, oncogenesis and hepatotoxicity. HepG2 was also considered representative of paediatric HCC since the donor was a 15-year-old boy. The histopathological background together with the original histology and recent molecular analyses have confirmed the HB characteristics of HepG2 [17], [18]. Hep3B cells were isolated from a young donor with HCC. The background of a HBV infection in these patients is obvious, as the Hep3B cells expressed constitutive HBsAg [12]. In contrast to Hep3B, the primary tumour of the HC-AFW1 line emerged from a background without any infections, which is the clinical situation in most cases of paediatric HCC in Europe.

In the case presented here, an extensive histological analysis of the original tumour, of the HC-AFW1 cell line, and of the derived xenografts was performed. Consensus reading by several international institutions classified the tumour as a HCC. Distinguishing between HB and paediatric HCC can sometimes be challenging. Occasionally, HCC-like foci have been postulated in HB post-chemotherapy as a result of a morphological maturation mimicking HCC [19]. Furthermore, the simultaneous presence of both tumour entities (HB/HCC) within the same child has also been reported and is referred to as transitional liver cell tumours (TLCT) [20]. TLCT develops in an age group older than that associated with the typical HB manifestation period and shows an aggressive behaviour. Neoadjuvant therapy may reduce the burden of HB, which is more sensitive to cytostatic agents than HCC. However, some HB characteristics, such as osteoid deposition and neuromelanin accumulation, persist after chemotherapy and can support the diagnosis of HB. The morphological appearance of the original tumour in our case was that of an HCC throughout; there were no HB-like areas within the tumour.

Efforts to characterize the HC-AFW1 cell line revealed a unique cytogenetic pattern including the isochromosome 1q, an interstitial deletion 5q, loss of chromosome 21, and a derivative chromosome 11. Whereas in HB gain of chromosome 1 and in HCC loss of 1p and 1q occur, HC-AFW1 showed obvious isochromosome 1q formation, leading to a loss of complete 1 p and a gain of 1q (confirmed by array data). In the remaining chromosome 1, cytogenetic analysis revealed an interstitial deletion of 1q32–43, which was not seen in the array. Additionally, the HC-AFW1 line showed a gain of terminal 2q and a gain of 22q, both typically seen in HB, but not in HCC. Loss of 4q—seen in both HB and HCC—was also found in HC-AFW1. Interestingly, an unbalanced translocation between chromosome 4 and 2q resulted in this deletion. In adult HCC, loss of 6q, 8p, 9p, 13q, 16p, 16q and 17p occur [21]. On the other hand, gain of chromosomes 7, 8, 17 and 20 is frequently seen in HB [22]. None of the latter anomalies were detected in HC-AFW1. Based on the cytogenetic analysis, HC-AFW1 appears to be biologically different from HB and from adult HCC. Therefore, the morphological assignment of HC-AFW1 as paediatric HCC is emphasized biologically. This again seems to underline the biological difference between paediatric and adult HCC.

Markers of liver tumours, such as Glypican-3, AFP and HepPar1, were present in HC-AFW1. The HC-AFW1 cell line also expressed epithelial cell markers such as E-Cadherin, CD326 and cytokeratins as well as Vimentin, CD44 and CD133, proteins that are often found in epithelial and mesenchymal tumours. An exact and definite assignment of paediatric liver tumours is not feasible based on expression markers alone due to the lack of exclusively specific markers for HB and HCC. HB may be distinguished from adult HCC by the expression of a panel of 11 genes [23]. However, there is no such panel to distinguish between paediatric HCC and HB. The most important contribution to diagnosing paediatric epithelial liver tumours thus remains the morphological analysis. Based on tumour morphology and clinical data, the consensus of the international pathological evaluation postulated paediatric HCC as the origin of the HC-AFW1 cell line.

HC-AFW1 cells are similar to the parental HCC cells in terms of the unique and conserved β-catenin deletion within the tumour. This deletion involves the phosphorylation site of GSK3beta, a region associated with preventing degradation and enhanced accumulation of β-catenin in the cell, and thus leads to excessive Wnt/β-catenin signalling. The CTNNB1 deletion is somatic and appears to affect only 1 of the 2 CTNNB1 alleles; the constitutional DNA showed no alterations. This denotes clonal development of this multinodular HCC. Large deletions spanning exon 3 in CTNNB1 are observed only sporadically in adult HCC [24] but are more common in HB and in childhood HCC [25]. Instead of being localized along the cytoplasmic membrane, β-catenin is strongly accumulated in the cytoplasm and nucleus; however, it is not evenly distributed in the tumour tissue. This accumulation of β-catenin provides a growth advantage to tumour cells by promoting proliferation and suppressing differentiation [26], [27]. β-catenin accumulation alone, however, does not seem to cause progression to HCC from a non-malignant state [28].

Overall, there was no hint of anaplastic differentiation however a selection during the culture process was observed. The stability of the cell line was supported by repeated cytogenetic analysis at different passages and by cytology. The constant expression pattern of selected tumour proteins as well as tumour uptake and growth rates in mice rendered HC-AFW1 a consistent *in vitro* and *in vivo* model of paediatric HCC.

In concordance with the clinically observed response to CDDP therapy, HC-AFW1 cells also showed chemosensitivity to CDDP. Other drugs targeting cell proliferation also affected the viability of HC-AFW1 cells. The drug concentrations required for 50% inhibition of cell culture viability were comparable with those observed in the treatment of HB [29]. HC-AFW1 seems to be a non-responder to inhibitors of microtubule assembly, such as vincristine, which is comparable to adult HuH7 HCC cells and occurs despite the high doubling time of the cells. Vincristine is a potent inhibitor of cell proliferation in most HCC-derived cell lines (IC50 at ca. 10 to 20 ng/ml) except for HuH7, which has an IC_50_ of 20 µg/ml [30]. Other cytotoxic drugs such as cisplatin, etoposide and carboplatin, have a heterogeneous impact on adult-derived HCC cell lines. However, HCC *in vivo* remains chemotherapy refractive to a high degree [31], [32]. This may result from the tumour architecture *in vivo* and the presence of tumour stem cells, which reduces responsiveness to drugs. A xenograft tumour model might help to further assess these factors and facilitate the development of treatment regimens. HC-AFW1 showed aggressive and robust growth in immune incompetent mice. All mice developed tumours within 4 weeks after transplantation of a relatively low number of tumour cells. This may be due to the selection of more proliferating cells with a nuclear distribution of β-catenin, of longer telomeres and of the high number of CD133-positive cells, which are considered to be tumour initiating [27]. The sustained proliferation and selection of cultured cells with longer telomeres and reduced senescence were also observed in conditions of active pathways like STAT3 [33]. When tumour fragments were used instead of cultured cells for xenotransplantation, growing tumours were observed subcutaneously within 10 days. This tendency of tumour cell adaptation to skin niches might be useful for further study of more aggressive tumour growth.

The HC-AFW1 cell line resembled parts of the original paediatric epithelial liver tumour and showed characteristics of HCC. The stable culture of HC-AFW1 and its high tumour incidence in immunodeficient mice are valuable for investigating the biology of and therapeutic strategies for childhood HCC.

## Supporting Information

Figure S1 **Expression of tumour-related proteins in HC-AFW1.** Images show immunofluorescence staining in primary tumour samples (upper rows) and subcutaneous xenografts (low rows). Red fluorescence denotes homogenous expression of CD90, CD326, cytokeratin type 1 (CK AE1) and HepPar1 as well as heterogeneous distribution of CD10, CD133, Vimentin and Cytokeratin 7 (CK7) within the tumour. Blue fluorescence indicates nuclei (DAPI staining). Bars represent 50 µm.
(TIF)

Figure S2 **Serum AFP level in mice bearing HC-AFW1 xenografts.** Seven days post-subcutaneous injection, HC-AFW1 cells led to increased serum AFP levels in NSG mice (n = 4). Sera were positive for AFP in all mice, although no tumours with a mean diameter >3 mm were observed initially.
(TIF)

Figure S3 **Deletion analysis of β-catenin gene in HC-AFW1 cells.** mRNA (A) and DNA (B) from liver (2) native tumor tissue (3), xenograft HC-AFW1 tissue (4) and from HC-AFW1 cells (5) was amplified by RT-PCR using the ctnb1for and rev primer for beta catenin (11). Expected RT-PCR products derived from β-catenin (833 bp) and the smaller product were isolated from the agarose gel and sequenced using the same primers. (1) Length marker of 100 bp ladder. (C) Sequence alignment of the larger PCR fragment (6975066) and of the smaller PCR fragment (6975070) with the published sequence for beta catenin (AY081165.1). The smaller product revealed a deletion of 147 nucleotides. Numbers denote the position in the sequence AY081165.1, (∼) represent gaps in alignment.
(TIF)

Figure S4 **Telomere length and senescence in HC-AFW1 cells.** HC-AFW1 cells at the indicated passages were analysed to determine telomere length using flow FISH (A). Senescent cells were detected by blue staining of acid beta galactosidase (B). Cells at lower passages had shorter telomeres and more were senescent compared with cells at higher passages.
(TIF)

Table S1 **Antibodies for immunohistological staining.**
(DOC)
