# Supplementary figures and images for: Characterisation of the Cell Line HC-AFW1 Derived from a Pediatric Hepatocellular Carcinoma
Source: PLoS One. 2012 May 30;7(5):e38223. doi: 10.1371/journal.pone.0038223 (PMC3364222; doi:10.1371/journal.pone.0038223)

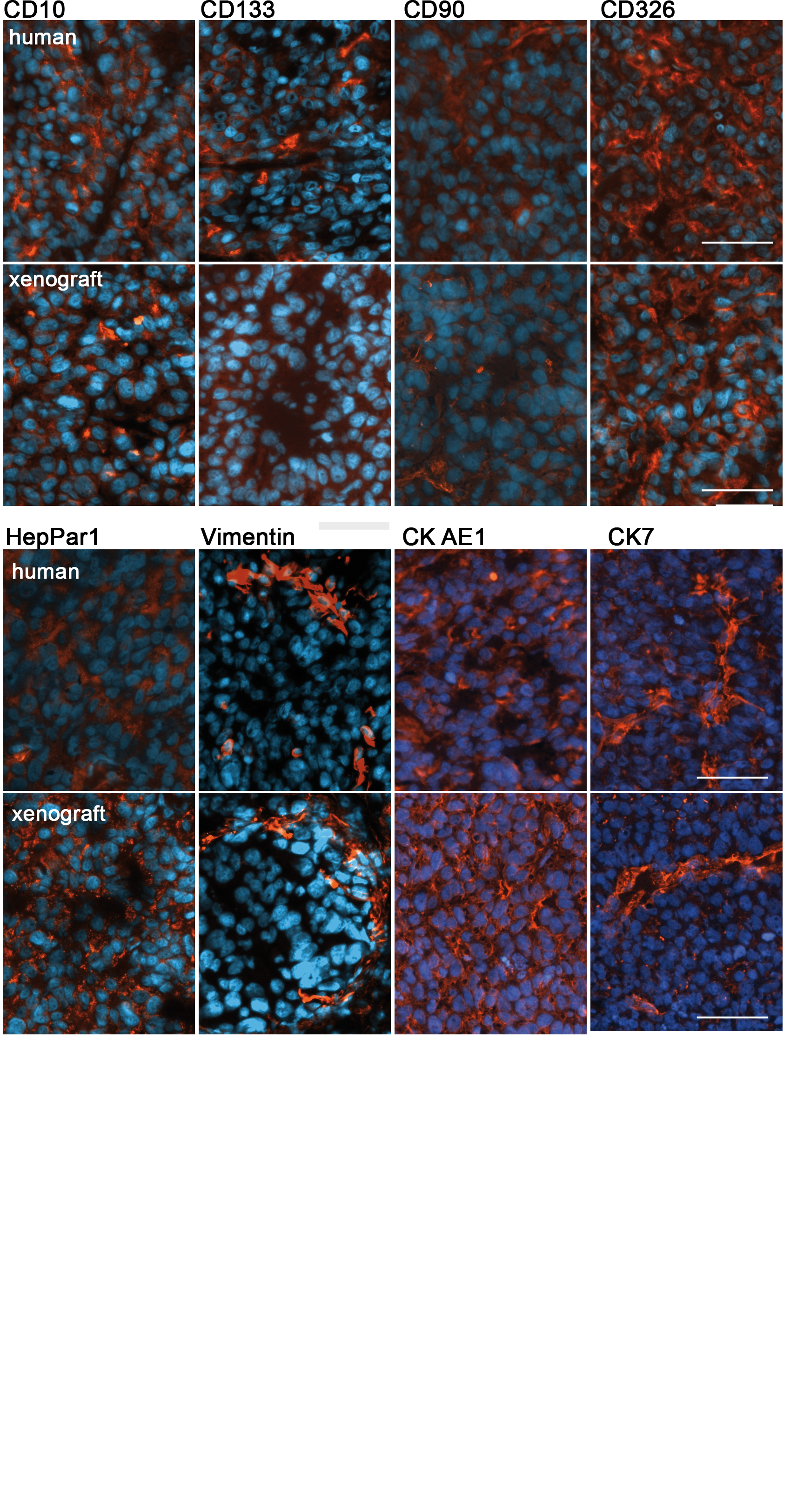

Supplement: Figure S1 — Expression of tumour-related proteins in HC-AFW1. Images show immunofluorescence staining in primary tumour samples (upper rows) and subcutaneous xenografts (low rows). Red fluorescence denotes homogenous expression of CD90, CD326, cytokeratin type 1 (CK AE1) and HepPar1 as well as heterogeneous distribution of CD10, CD133, Vimentin and Cytokeratin 7 (CK7) within the tumour. Blue fluorescence indicates nuclei (DAPI staining). Bars represent 50 µm. (TIF) [file pone.0038223.s001.tif]

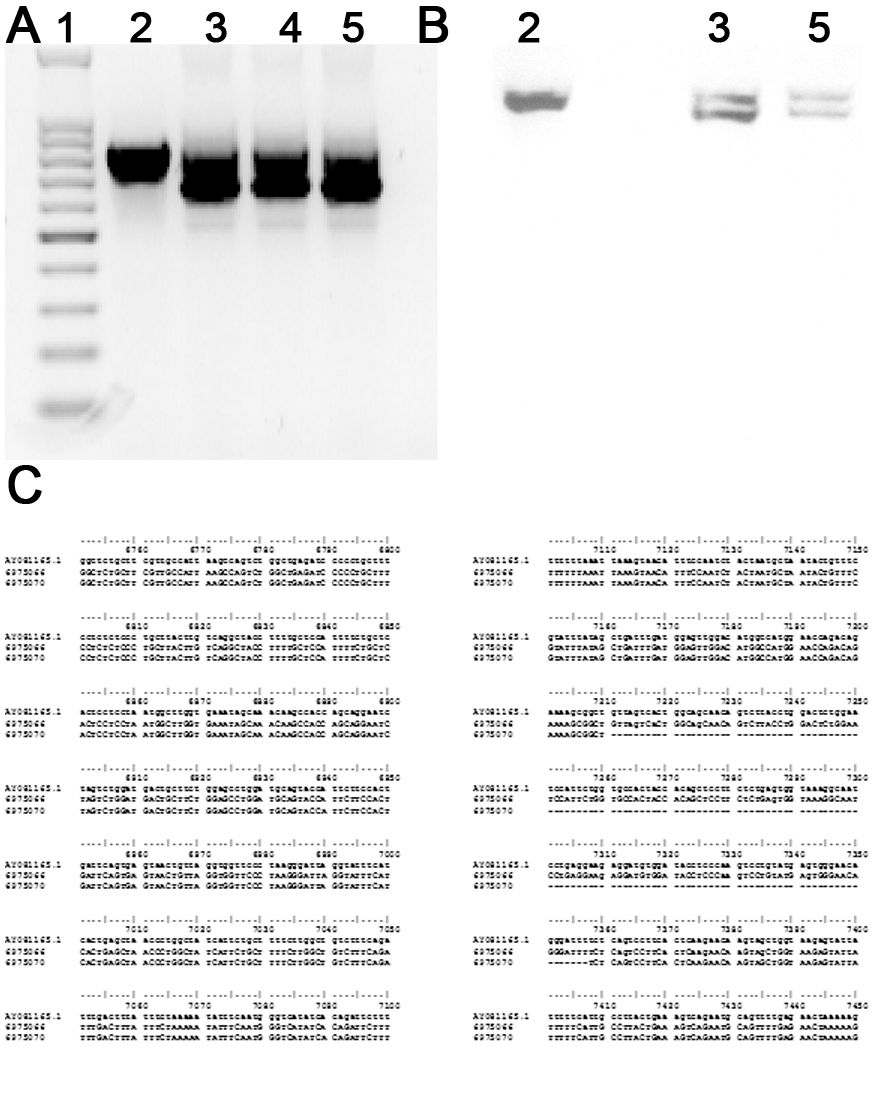

Supplement: Figure S3 — Deletion analysis of β-catenin gene in HC-AFW1 cells. mRNA (A) and DNA (B) from liver (2) native tumor tissue (3), xenograft HC-AFW1 tissue (4) and from HC-AFW1 cells (5) was amplified by RT-PCR using the ctnb1for and rev primer for beta catenin (11). Expected RT-PCR products derived from β-catenin (833 bp) and the smaller product were isolated from the agarose gel and sequenced using the same primers. (1) Length marker of 100 bp ladder. (C) Sequence alignment of the larger PCR fragment (6975066) and of the smaller PCR fragment (6975070) with the published sequence for beta catenin (AY081165.1). The smaller product revealed a deletion of 147 nucleotides. Numbers denote the position in the sequence AY081165.1, (∼) represent gaps in alignment. (TIF) [file pone.0038223.s003.tif]

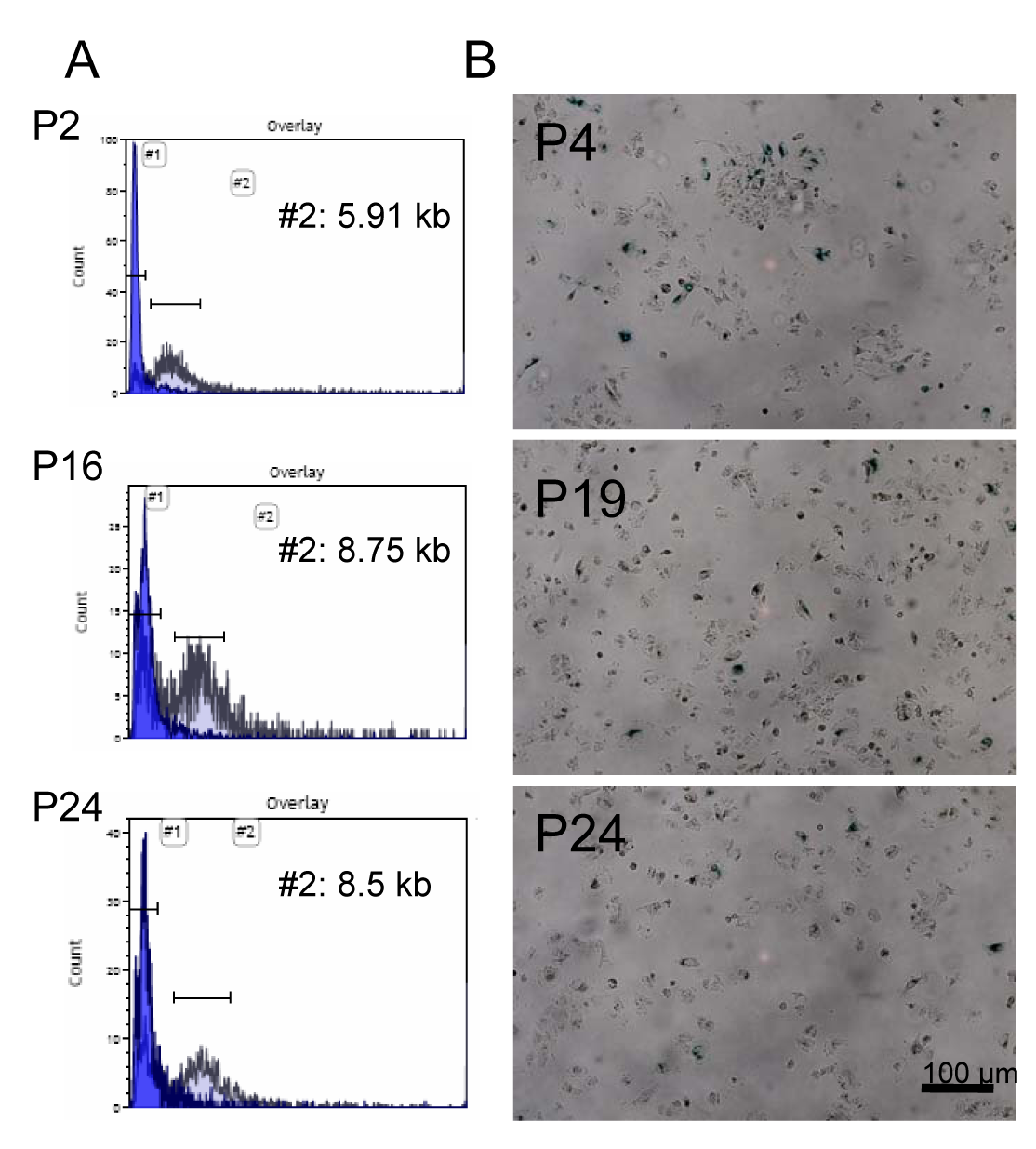

Supplement: Figure S4 — Telomere length and senescence in HC-AFW1 cells. HC-AFW1 cells at the indicated passages were analysed to determine telomere length using flow FISH (A). Senescent cells were detected by blue staining of acid beta galactosidase (B). Cells at lower passages had shorter telomeres and more were senescent compared with cells at higher passages. (TIF) [file pone.0038223.s004.tif]
