# Supplementary material for: Characterisation of the Cell Line HC-AFW1 Derived from a Pediatric Hepatocellular Carcinoma
Source: PLoS One. 2012 May 30;7(5):e38223. doi: 10.1371/journal.pone.0038223 (PMC3364222; doi:10.1371/journal.pone.0038223)
Supplement: Table S1 — Antibodies for immunohistological staining. (DOC) [file pone.0038223.s005.doc]

**Table S1: Antibodies for immuno**histological staining.

| **Antigen** | **Origin** | **Clone** | **Distributor** | **Dilution** | **Sec. Antibody** |
| --- | --- | --- | --- | --- | --- |
| CD10 | Mouse | 97C5A6 | Dr. Bühring | 1:5 | 1 |
| CD90 | Mouse | Ebio5E10 | eBiosciense | 1:20 | 1 |
| Vimentin | Mouse | Vim3b4 | DAKO | 1:100 | 1 |
| CD326 | Human | HEA-125 | Miltenyi Biotec | 1:100 | 1 |
| β-catenin | Mouse | 14/Β-catenin | BD | 1:100 | FITC conj. |
| Ecadherin | Mouse | 67A4 | Dr. Bühring | 1:5 | 1 |
| CD133 | Mouse | W6B3H10 | Dr. Bühring | 1:5 | 1 |
| CD44 | Mouse | IM7 | eBiosciense | 1:20 | PE conj. |
| Cytokeratin type 1 | Mouse | AE1/AE3 | DAKO | 1:100 |  |
| Cytokeratin 7 | Mouse | OVTL12/30 | DAKO | 1:100 |  |

1: Alexa Fluor 546 goat anti-mouse IgG PE (1:500)
